# Chitinase‐3‐Like 1 Protein (CHI3L1) Levels in Patients With Cognitive Deficits and Movement Disorders: Comparison With Other Biomarkers

**DOI:** 10.1002/brb3.70619

**Published:** 2025-06-10

**Authors:** R. Novobilský, P. Bártová, D. Stejskal, A. Kondé, M. Bar, P. Kušnierová

**Affiliations:** ^1^ Department of Neurology University Hospital Ostrava Ostrava Czech Republic; ^2^ Department of Clinical Neurosciences University of Ostrava Ostrava Czech Republic; ^3^ Institute of Laboratory Medicine University of Ostrava Ostrava Czech Republic; ^4^ Institute of Laboratory Medicine, Department of Clinical Biochemistry University Hospital Ostrava Ostrava Czech Republic; ^5^ Faculty of Electrical Engineering and Computer Science, Department of Applied Mathematics VSB–Technical University of Ostrava Ostrava Czech Republic; ^6^ Department of the Deputy Director for Science, Research, and Education University Hospital Ostrava Ostrava Czech Republic

**Keywords:** Alzheimer's disease, biomarkers, CHI3L1, dementia

## Abstract

**Introduction:**

Chitinase‐3‐like protein 1 (CHI3L1) is a glycoprotein implicated in various neurological conditions. It is associated with neuroinflammation and tissue remodeling. The study aimed to validate the reference interval (RI) of serum (S) CHI3L1 in a control group, to correlate S CHI3L1 values with other biomarkers of neurodegenerative damage, and to estimate the diagnostic accuracy of S CHI3L1.

**Methods:**

Samples from 108 healthy volunteers were used to estimate the S CHI3L1 RI. For the comparison, we used cerebrospinal fluid (CSF) and serum (S) samples from 121 patients with cognitive disorders, and cognitive deterioration was assessed using the Mini‐Mental State Examination (MMSE). ELISA assays were used to determine the S CHI3L1, CSF, and S neurofilament light chain (NfL) levels; CSF and plasma β‐amyloid peptide_42_; CSF and plasma β‐amyloid peptide_40_; CSF total tau protein; CSF phosphorylated tau protein; and CSF alpha‐synuclein.

**Results:**

The estimated RI of S CHI3L1 was 14.44 to 63.11 µg/L. The cut‐off value of S CHI3L1 was 34.37 µg/L. ROC analysis showed that S CHI3L1 has 81.4% sensitivity and 76.9% specificity. We found a moderate Spearman's rank correlation coefficient between the S CHI3L1 and age (*r*_S _= 0.486; *p* < 0.001) and between S CHI3L1 and S NfL (*r*_S_ = 0.489; *p* < 0.001) in all groups. The Kruskal–Wallis test showed a significant overall difference in S CHI3L1 among diagnostic groups (*p* = 0.013). S CHI3L1 and CSF NfL had statistically significant effects on MMSE values (multiple *R* ^2^ was 0.431).

**Conclusions:**

Our results suggest that S CHI3L1 reflects the severity of cognitive deficits assessed by MMSE. It can be used as a supportive biomarker in neurodegenerative diseases.

## Graphical abstract

This study examines chitinase‐3‐like 1 protein (CHI3L1) as a diagnostic tool for patients with cognitive and movement disorders. Serum CHI3L1 levels correlate with neurofilament light chain and cognitive impairment measured by the Mini‐Mental State Examination (MMSE). CHI3L1 may serve as a supportive biomarker in these diseases.

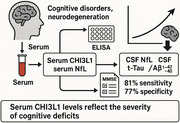

## 1 Introduction

Neurodegenerative diseases are a broad group of nervous system disorders characterized by the progressive and irreversible loss of specific neuron populations in both the central and the peripheral nervous systems. This leads to a variety of clinical symptoms, commonly including dementia and movement disorders. Research suggests that neurodegeneration may be caused by the accumulation of specific proteins in the tissues, resulting in inflammation and cell death (Ciccocioppo et al. 2020; Dugger and Dickson 2017).

These diseases are most prevalent among individuals over 65 years of age, and their occurrence rises with age. It is estimated that the number of individuals with dementia will double in the next 30 years due to increasing life expectancy (Ferri et al. 2005; Nichols et al. 2019). Therefore, laboratory biomarkers are sought to aid in the early diagnosis of neurodegenerative diseases, with the hope of a potential cure in the future. Thanks to the investigation of Alzheimer's disease (AD), we have a great knowledge of the classical neurodegenerative biomarkers‐beta‐amyloid_42_ (Aβ_42_), total tau protein (tTau), phosphorylated tau protein_181_ (pTau), and, more recently, neurofilament light chain (NfL) (Blennow and Zetterberg 2018). As in all other processes in life, inflammation plays a role in neurodegeneration. According to recent studies, it seems that inflammation in the central nervous system is not simply a response to hallmark pathologies but is itself pathogenic (Connolly et al. 2023).

Chitinase‐3‐like protein 1 (CHI3L1, or YKL‐40) is a secreted glycoprotein that belongs to the diverse glycoside hydrolase family 18 (Rehli et al. 1997). It is a known biomarker of immune response and inflammation in peripheral tissue and is expressed by macrophages, chondrocytes, osteocytes, epithelial and endothelial cells, smooth muscle cells, hepatic cells, tumor cells, and so forth. It influences cell division and survival, inhibits apoptosis, stimulates tissue remodeling, and contributes to immune cell activation. In the brain, it is expressed mainly in astrocytes, especially in reactive neurotoxic astrocytes induced by microglia (Bonneh‐Barkay et al. 2012; Connolly et al. 2023; Lee et al. 2011; Sutherland 2018).

Many studies have demonstrated that the concentration of CHI3L1 in CNS, CSF, and plasma in AD patients is elevated, and according to some of them, it can help differentiate AD from frontotemporal dementia (FTD) (Hampel et al. 2018). Similarly, the concentrations of other molecules from the chitinase family (e.g., chitotriosidase, acidic mammalian chitinase, chitinase 3‐like protein 2) are increased in the brains of AD patients (Sanfilippo et al. 2016). In the recently published study by Pase et al. (2024), CHI3L1 was considered a nonspecific prognostic biomarker of cognitive impairment independent of AD processes. As with other biomarkers of neurodegeneration, CHI3L1 is considered a potential therapeutic target for future trials (Yu et al. 2024).

The study aimed to validate the reference interval (RI) for serum chitinase 3‐like 1 (S CHI3L1) in a control group and measure the CHI3L1 concentration in different diagnostic groups, including individuals with AD, non‐AD dementia, Parkinson's disease, other movement disorders, a combination of a cognitive syndrome and a movement disorder, and healthy controls. Additionally, the study sought to correlate these values with other biomarkers of neurodegenerative damage in different groups and to estimate the diagnostic accuracy of S CHI3L1.

## 2 Materials and Methods

### 2.1 Patients and Data Collection

To determine the reference values for S CHI3L1, serum samples were collected from healthy adults from the Blood Centre of the University Hospital Ostrava (*n* = 108, average age 43.6 ± 10.1 years; 53 females, average age 42.6 ± 9.7 years; 55 males, average age 44.8 ± 10.5 years). With the exception of sex and age, all other patient data was anonymized. All volunteers exhibited good health and did not report any medication usage.

For comparative analysis, we collected cerebrospinal fluid, EDTA blood, and serum samples from patients at the University Hospital Ostrava, Czech Republic, who were included in a single‐center prospective cohort study (*n* = 121), 71 female patients (58.7%) and 50 male patients (41.3%). The samples were collected from May 1, 2021, until June 30, 2023. The cerebrospinal fluid, collected through a lumbar puncture in intervertebral space L3/L4, L4/5, or L5/S1, had a standardized volume of 10 ± 1 mL from all patients.

The inclusion criteria were as follows: (1) signed written informed consent for study inclusion; (2) brain imaging (CT or MRI) performed to exclude space‐occupying brain lesions such as tumors, brain contusions, multiple sclerosis, normal‐pressure hydrocephalus, and large postischemic or posthemorrhagic lesions; (3) laboratory examinations performed to exclude other causes of cognitive deficit, such as ion imbalance, anemia, B12 hypovitaminosis, Wilson's disease, and thyroid disorder; (4) A cognitive screening conducted utilizing a Mini‐Mental State Examination (MMSE); (5) a neurodegenerative movement disorder, with primary complaints other than dementia (Parkinson's disease, multiple system atrophy, progressive supranuclear palsy, etc.).

The exclusion criteria were as follows: (1) an age lower than 18 years; (2) space‐occupying brain lesion displayed by MRI/CT; (3) metabolic etiology of cognitive deficit as mentioned above.

The control group comprised patients with an MMSE score of > 28/30 and no clinical signs of Parkinsonism (including tremor, rigidity, bradykinesia, hypokinesia, and gait disturbance) as assessed by an experienced neurologist. Dementia was defined as an MMSE score of ≤ 25/30 points, with a temporal aspect of at least 6 months of clinical symptoms affecting daily activities. The cut‐off score of 25 points on the MMSE was established due to limitations on reimbursement by Czech insurance for AD treatment. One patient with severe dementia was unable to complete the MMSE due to noncooperation.

The patients were divided into five groups based on their clinical characteristics and neuropsychological assessments. Group 1 consisted of individuals with AD, as defined by the National Institute on Aging and Alzheimer's Association (NIA‐AA) research criteria for AD (Jack et al. 2018) (*n* = 36; average age 71.3 ± 9.1 years). Group 2 included subjects diagnosed with non‐Alzheimer's dementia (*n* = 33; average age 70.8 ± 10.0 years). Group 3 comprised individuals diagnosed with Parkinson's disease and patients with movement disorders without cognitive deficit (*n* = 24; average age 62.8 ± 10.9 years). Group 4 comprised individuals diagnosed with a combination of a cognitive syndrome and a movement disorder (*n* = 10; average age 67.8 ± 13.1 years). Group 5 consisted of healthy controls (*n* = 18; average age 58.1 ± 11.5 years). In Group 1, the diagnoses included 6 cases of AD according to the NIA‐AA research criteria for AD without dementia, 12 cases of AD according to the NIA‐AA research criteria for AD, and 18 cases of AD diagnosed by an experienced neurologist when patients declined lumbar puncture. In group 2, non‐Alzheimer dementias comprised 16 cases of vascular dementia, 8 cases of FTD, 3 cases of Lyme neuroborreliosis, 3 cases of alcohol‐related dementia, 2 cases of Creutzfeldt–Jakob disease (CJD), and 1 case of primary progressive aphasia. In group 3, the diagnoses included 14 cases of Parkinson's disease diagnosed by an experienced neurologist according to the criteria of the Movement Disorder Society (Postuma et al. 2015) and 10 cases of patients with movement disorders other than Parkinson's disease without dementia, including multiple system atrophy (*n* = 4), progressive supranuclear palsy (*n* = 2), dystonia (*n* = 2), Huntington's disease (*n* = 1), and essential tremor plus syndrome (*n* = 1). Group 4, which comprised patients with a combination of a cognitive syndrome and a movement disorder, consisted of 6 cases of Lewy body disease, 2 cases of multiple system atrophy, 1 case of spinocerebellar ataxia, and 1 case of progressive supranuclear palsy.

### 2.2 Samples

An atraumatic needle was used to obtain all CSF samples placed in polypropylene tubes (Sarstedt, Nümbrecht, Germany). Serum samples were collected in tubes with Serum Gel and Clotting Activator (Sarstedt). Subsequently, the CSF samples were centrifuged at 390 × *g* for 10 min at room temperature, and the serum samples were centrifuged at 2500 × *g* for 6 min at 4°C. Following centrifugation, the CSF and serum samples were divided into at least three vials containing 0.3 mL and stored at −70°C until analysis. Concurrently, blood was collected with the addition of ethylenediaminetetraacetic acid (EDTA; Sarstedt) to determine the complete blood count (CBC), which includes the total leukocyte (white blood cell [WBC]) count.

### 2.3 Analytical Methods

The concentrations of S CHI3L1 were determined by ELISA (Human Chitinase 3‐like 1 Immunoassay, REF DC3L10, RUO, R&D Systems, Inc.). The kit manufacturer stated that the detection limit was 3.55 ng/L. All samples were analyzed in duplicate. Measurements of S CHI3L1 were performed using a 50‐fold diluted serum.

The number of leukocytes (WBC) was evaluated as a hematological parameter on a Sysmex XN‐9000. The levels of CRP were analyzed on an Atellica CH Analyzer with an Atellica CH C‐reactive protein_2 (CRP_2) kit produced by Siemens Healthcare Diagnostics Inc.

Concentrations of other neurodegenerative biomarkers were determined by ELISA methods using diagnostic kits: Total‐Tau‐ELISA, REF. EQ 6531‐9601‐L; Beta‐Amyloid (1‐42)‐ELISA, REF. EQ 6521‐9601‐L; Beta‐Amyloid (1‐40)‐ELISA, REF. EQ 6511‐9601‐L; pTau(181) ELISA, REF EQ‐6591‐9601‐L (Euroimmun); Alpha‐Synuclein ELISA, REF EQ 6545‐9601‐L; NF‐light ELISA CE, REF. 10–7001; NF‐light Serum ELISA RUO, REF. 20–8002, Uman Diagnostics; Plasma Beta‐Amyloid (1‐42)‐ELISA, REF. EQ 6521–9601; plasma Beta‐Amyloid (1‐40)‐ELISA, REF. EQ 6511–9601.

### 2.4 Statistical Methods

Numerical variables are presented as medians, means, standard deviations (SD), and ranges (minimum and maximum), and categorical variables are presented as absolute and relative frequencies (%). The significance of between‐group differences was tested with the Mann–Whitney test or Kruskal–Wallis test. Assessment of relationships of numerical variables was performed using Spearman's correlation coefficient, its significance test, and stepwise multinomial linear regression analysis. The diagnostic accuracy of CHI3L1 was assessed with ROC analysis and evaluated with sensitivity and specificity and corresponding confidence intervals. The statistical analysis was performed in R software (version 4.3.1), and the significance level was set to 0.05.

### 2.5 Ethics Approval

Written informed consent was obtained from all included patients at the University Hospital Ostrava. The University Hospital Ostrava Ethics Committee approved the study as part of the project “Laboratory biomarkers of neurodegenerative diseases” (reference number 340/2021).

## 3 Results

### 3.1 Estimation of the CHI3L1 Reference Range With Control Group

**FIGURE 1:**
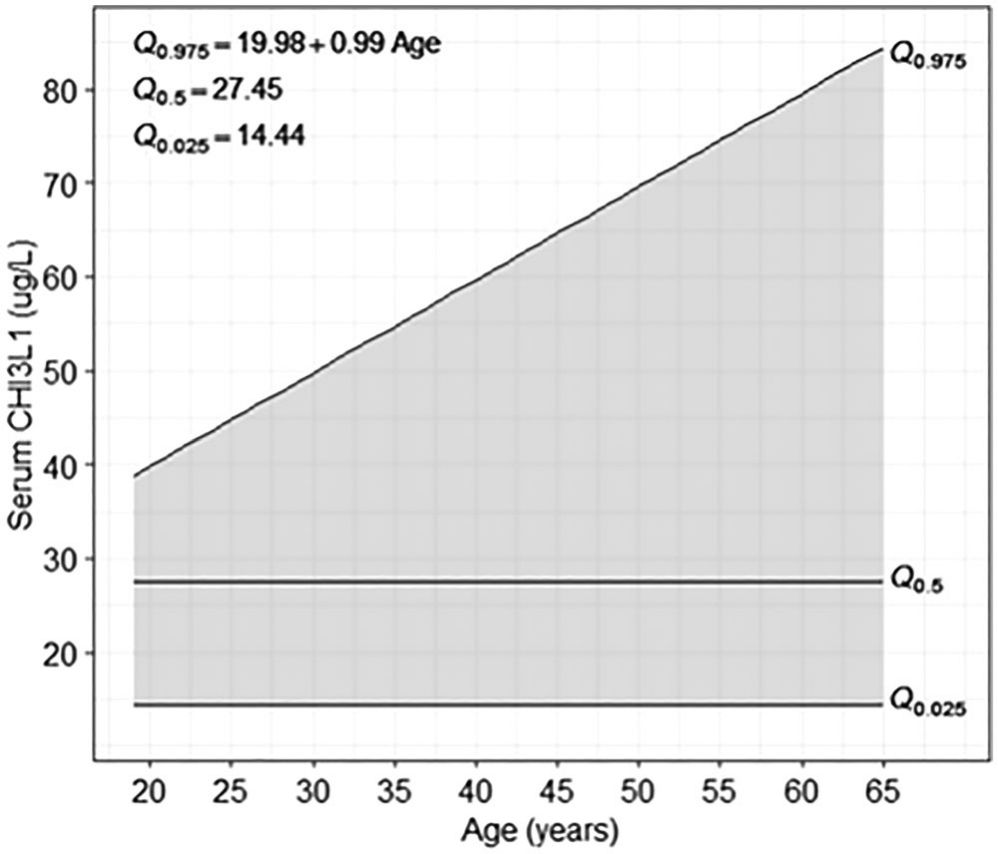
Estimation of the CHI3L1 reference range with the control group.

Due to the sample size of the control group (*n* = 108) and the non‐normal distribution of S CHI3L1 in the control group, a nonparametric approach for estimating the reference range was applied. Based on the 2.5% and 97.5% quantiles, the estimated reference range is 14.44–63.11 µg/L. With quantile regression, the dependence of the 2.5% and 97.5% quantiles on age and sex was examined. Based on the quantile regression model, we did not find a significant dependence of the 2.5% quantile of S CHI3L1 (i.e., the estimated lower bound of the reference range) on sex or age. However, we found a significant dependence of the 97.5% quantile of S CHI3L1 (i.e., the estimated upper bound of the reference range) on age: the corresponding quantile regression coefficient was statistically significant (β = 0.99; *p* < 0.001; Figure 1).

### 3.2 Patient Characteristics: All Patients

In total, 121 patients were included: 71 female patients (58.7%) and 50 male patients (41.3%). The median age of all patients was 69 years (±11.74 years), and the median age of female and male patients did not differ significantly (*p* = 0.277; Mann–Whitney test). Other analytical characteristics of the study population are presented in Table 1.

**TABLE 1:** Patient characteristics (*n* = 121 patients).

	n	Mean	Median	SD	Min	Max
Age (years)	121	67.2	69.0	11.4	36.0	88.0
CSF						
tTau (ng/L)	79	472	309	487	86.1	2748
pTau (ng/L)	79	73.3	41.2	80.8	1.50	376
Aβ 1–42 (ng/L)	79	1146	1127	529	123	2502
Aβ 140 (ng/L)	59	7852	7884	3211	357	15288
Aβ1‐42/Aβ1‐40	57	0.223	0.175	0.479	0.010	3.733
alpha‐synuclein (ng/L)	59	2836	2253	3583	19.0	28801
NfL (ng/L)	79	1832	1036	2049	239	10865
Plasma						
Aβ 1–42 (ng/L)	68	12.4	9.07	7.66	6.00	31.4
Aβ 1–40 (ng/L)	64	147	144	42.2	48.0	226
Aβ1‐42/Aβ1‐40	59	0.087	0.063	0.060	0.001	0.222
Serum						
NfL (ng/L)	106	26.2	20.3	23.2	2.30	168
CHI3L1 (µg/L)	86	124.9	58.7	160.2	18.2	745.4
CRP (mg/L)	83	5.06	4.00	6.61	0.18	45.8
Blood						
WBC (10^9^/L)	79	7.35	7.12	1.66	3.61	11.3

*Note*: The values represent the median and the interquartile range, or absolute and relative frequencies (%).

### 3.3 Analysis of Dependence of CHI3L1 and Other Selected Variables

We analyzed the dependence of serum biomarkers and other selected variables in Tables 2 and 3.

**TABLE 2:** Correlation analysis of serum CHI3L1 and age, cerebrospinal fluid (CSF) biomarkers, plasma and serum biomarkers, and WBC for all patients and each diagnostic group.

		All patients	Diagnostic group				
			1	2	3	4	5
Age	*r*_S_	0.486	0.394	0.565	0.547	—	0.196
	*p*	< 0.001	0.031	0.018	0.013	—	0.522
	*n*	86	30	17	20	6	13
CSF tTau	*r*_S_	0.341	0.255	0.238	0.327	—	−0.236
	*p*	0.019	0.379	0.582	0.327	—	0.514
	*n*	47	14	8	11	4	10
CSF pTau	*r*_S_	0.203	0.026	0.381	0.391	—	0.139
	*p*	0.170	0.929	0.360	0.237	—	0.707
	*n*	47	14	8	11	4	10
CSF Aβ 1–42	*r*_S_	−0.128	0.431	−0.310	0.309	—	0.006
	*p*	0.391	0.124	0.462	0.356	—	> 0.999
	*n*	47	14	8	11	4	10
CSF Aβ 1–40	*r*_S_	0.313	0.071	—	—	—	0.055
	*p*	0.086	0.882	—	—	—	0.892
	*n*	31	8	3	7	3	10
CSF Aβ1‐42/Aβ1‐40	*r*_S_	−0.383	−0.048	—	—	—	0.367
	*p*	0.041	0.935	—	—	—	0.336
	*n*	29	8	2	7	3	9
CSF alpha‐synuclein	*r*_S_	0.318	−0.095	—	—	—	−0.212
	*p*	0.082	0.840	—	—	—	0.560
	*n*	31	8	3	7	3	10
CSF NFL	*r*_S_	0.362	0.573	0.055	0.539	—	0.126
	*p*	0.013	0.071	0.892	0.113	—	0.700
	*n*	47	11	10	10	4	12
Plasma Aβ 1–42	*r*_S_	0.064	0.074	—	−0.467	—	0.546
	*p*	0.688	0.820	—	0.178	—	0.102
	*n*	42	12	6	10	4	10
Plasma Aβ 1–40	*r*_S_	−0.182	−0.030	—	−0.600	—	−0.345
	*p*	0.288	0.946	—	0.097	—	0.331
	*n*	36	10	3	9	4	10
Plasma Aβ1‐42/Aβ1‐40	*r*_S_	0.211	0.383	—	−0.048	—	0.455
	*p*	0.239	0.312	—	0.935	—	0.191
	*n*	33	9	3	8	3	10
Serum NFL (ng/L)	*r*_S_	0.490	0.415	0.407	0.577	—	−0.128
	*p*	< 0.001	0.028	0.133	0.011	—	0.725
	*n*	78	28	15	19	6	10
Serum CRP (mg/L)	*r*_S_	0.097	−0.312	−0.187	−0.370	—	0.459
	*p*	0.484	0.223	0.604	0.236	—	0.156
	*n*	54	17	10	12	4	11
Blood WBC (10^9^/L)	*r*_S_	−0.009	0.046	−0.418	0.077	—	−0.091
	*p*	0.950	0.871	0.265	0.812	—	0.803
	*n*	50	15	9	12	4	10

*Note*: The values represent Spearman's correlation coefficient (*r*_S_), the *p* value of its test of significance (*p*), and the number of patients (*n*).

**TABLE 3:** Correlation analysis of serum NFL and age, cerebrospinal fluid (CSF) biomarkers, plasma and serum biomarkers, WBC, and serum CHI3L1 for all patients and each diagnostic group.

		All patients	Diagnostic group				
			1	2	3	4	5
Age	*r*_S_	0.487	0.458	0.071	0.441	0.109	0.888
	*p*	< 0.001	0.008	0.714	0.040	0.763	< 0.001
	*n*	106	32	29	22	10	13
CSF tTau	*r*_S_	0.265	−0.025	0.120	0.468	0.167	0.697
	*p*	0.022	0.922	0.596	0.094	0.703	0.012
	*n*	74	18	22	14	8	12
CSF pTau	*r*_S_	0.200	−0.057	0.021	0.359	0.310	0.746
	*p*	0.088	0.823	0.928	0.208	0.462	0.005
	*n*	74	18	22	14	8	12
CSF Aβ 1–42	*r*_S_	0.005	−0.129	0.254	0.631	−0.238	0.224
	*p*	0.965	0.610	0.254	0.018	0.582	0.484
	*n*	74	18	22	14	8	12
CSF Aβ 1–40	*r*_S_	−0.074	−0.466	−0.127	0.370	—	0.077
	*p*	0.579	0.127	0.625	0.296	—	0.802
	*n*	59	12	17	10	7	13
CSF Aβ1‐42/Aβ1‐40	*r*_S_	0.027	0.042	0.250	−0.006	—	0.564
	*p*	0.843	0.897	0.349	> 0.999	—	0.056
	*n*	57	12	16	10	7	12
CSF alpha‐synuclein	*r*_S_	0.192	−0.343	0.348	0.273	—	0.382
	*p*	0.146	0.275	0.171	0.448	—	0.197
	*n*	59	12	17	10	7	13
CSF NFL	*r*_S_	0.729	0.341	0.556	0.835	0.881	0.902
	*p*	< 0.001	0.213	0.007	0.001	0.007	< 0.001
	*n*	72	15	23	13	8	13
Plasma Aβ 1–42	*r*_S_	0.032	0.141	0.314	0.225	−0.905	−0.074
	*p*	0.799	0.603	0.178	0.459	0.005	0.830
	*n*	68	16	20	13	8	11
Plasma Aβ 1–40	*r*_S_	0.148	0.339	−0.444	0.119	0.690	0.391
	*p*	0.243	0.236	0.076	0.716	0.069	0.187
	*n*	64	14	17	12	8	13
Plasma Aβ1‐42/Aβ1‐40	*r*_S_	−0.106	0.041	0.463	0.100	—	0.073
	*p*	0.425	0.894	0.063	0.776	—	0.831
	*n*	59	13	17	11	7	11
Serum CHI3L1	*r*_S_	0.490	0.415	0.407	0.577	—	−0.128
	*p*	< 0.001	0.028	0.133	0.011	—	0.725
	*n*	78	28	15	19	6	10
Serum CRP (mg/L)	*r*_S_	0.016	0.362	−0.060	−0.050	—	−0.173
	*p*	0.895	0.107	0.786	0.865	—	0.633
	*n*	74	21	23	14	6	10
Blood WBC (10^9^/L)	*r*_S_	0.006	−0.096	−0.003	−0.109	—	−0.644
	*p*	0.962	0.697	0.988	0.710	—	0.044
	*n*	72	19	23	14	6	10

*Note*: The values represent Spearman's correlation coefficient (*r*_S_), the *p* value of its test of significance (*p*), and the number of patients (*n*).

**FIGURE 2:**
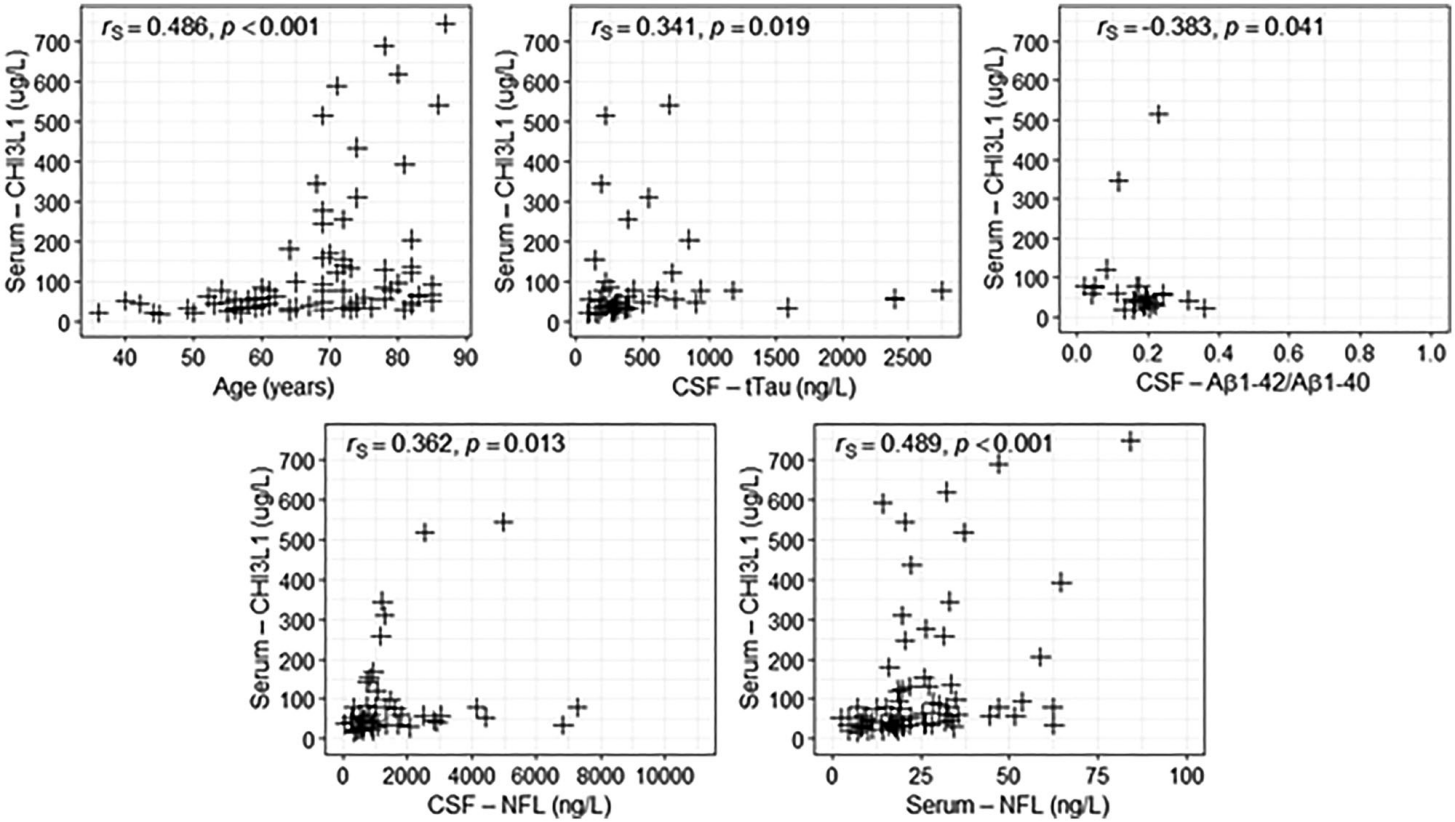
Visualization of significant correlations of serum CHI3L1 and selected variables.

In all groups, we found a moderate Spearman's rank correlation coefficient between S CHI3L1 and age (*r*_S _= 0.486; *p* < 0.001) and between S CHI3L1 and S NfL (*r*_S_ = 0.489; *p* < 0.001). We found a weak statistically significant correlation between S CHI3L1 and CSF tTau (*r*_S_ = 0.341; *p* = 0.019), between S CHI3L1 and CSFAβ1‐42/Aβ1‐40 (*r*_S_ = −0.383; *p* = 0.041), and between S CHI3L1 and CSF NfL (*r*_S_ = 0.362; *p* = 0.013) in all groups together. A weak correlation was observed between S CHI3L1 and age in Group 1 (*r*_S_ = 0.394; *p* = 0.031). Moderate correlation coefficients were found between S CHI3L1 and age (*r*_S _= 0.565; *p* = 0.018) in Groups 2 and 3 (*r*_S _= 0.547; *p* = 0.013), and between S CHI3L1 and S NfL in Groups 1 (*r*_S_ = 0.415; *p* = 0.028) and 3 (*r*_S_ = 0.577; *p* = 0.011) (Figure 2).

No significant difference was found between female and male patients in medians of S CHI3L1 and medians of S NFL (*p* = 0.779, *p* = 0.281, Mann–Whitney test).

### 3.4 Analysis of the Dependence of Serum Biomarkers and Diagnostic Groups

**FIGURE 3:**
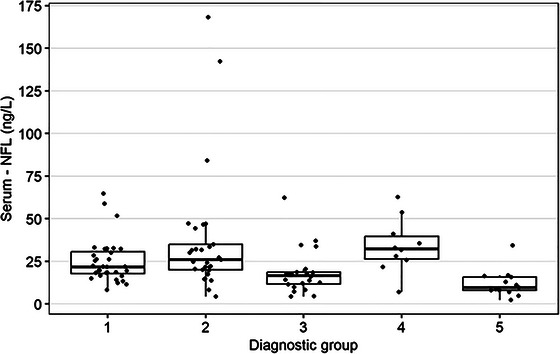
Visualization of the comparisons of the diagnostic groups’ serum NFL (ng/L).

**FIGURE 4:**
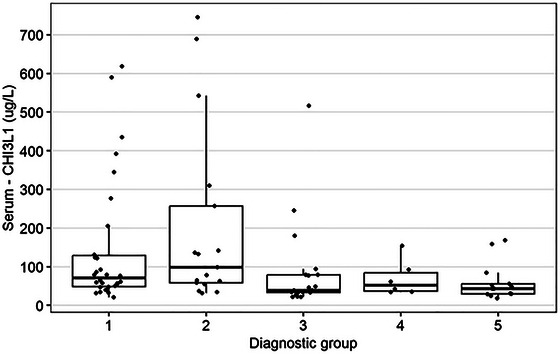
Visualization of the comparisons of the diagnostic groups’ serum CHI3L1 (µg/L).

The Kruskal–Wallis test showed a significant overall difference among diagnostic groups (see Table 4, Figures 3 and 4). However, the post hoc Dunn test, employed to identify specific between‐group differences, did not show any significant results, suggesting caution in interpreting individual group differences.

**TABLE 4:** Comparisons of diagnostic groups’ serum NFL and serum CHI3L1.

	Serum NFL (ng/L)		Serum CHI3L1 (µg/L)	
	*n*	Median (Min; Max)	*n*	Median (Min; Max)
Diagnostic group				
1	32	21.8 (8.3; 64.7)	30	70.9 (20.9; 618.9)
2	29	25.9 (4.4; 168.2)	17	98.8 (31.3; 745.4)
3	22	16.6 (4.4; 62.3)	20	39.1 (21.8; 516.0)
4	10	32.3 (7.0; 62.6)	6	51.9 (34.5; 154.1)
5	13	9.6 (2.3; 34.4)	13	43.4 (18.2; 168.8)
*p*‐value of Kruskal–Wallis test		< 0.001	0.013	
Homogeneous groups (Dunn's post hoc analysis)		(4, 2, 1), (1, 3), (3, 5)	No significant differences were found.	

### 3.5 Stepwise Multinomial Linear Regression Analysis

We conducted a stepwise multinomial linear regression analysis to evaluate the impact of biochemical markers on MMSE values as a marker of disease progression. We found statistically significant effects of S CHI3L1 and CSF NfL (Table 5). The multiple *R* ^2^ value was 0.431, the adjusted *R* ^2^ was 0.393, and the *F* statistic was 11.354 at 2 and 30 DF (*p *< 0.001). These results indicate that these two variables can explain 43.1% of the MMSE values' variability.

**TABLE 5:** Final stepwise multinomial linear regression analysis: MMSE = 0.453 + 4.25*10^−6^ × 1S CHI3L1 + 4.40 × 110^−4^ × 1CSF NfL; Multiple *R* ^2^ = 0.431.

Coefficients	Estimate	Std. error	*t* value	*p* (> |t|)
S CHI3L1	4.25 × 10^−6^	1.89 × 10^−6^	2.247	0.032
CSF NfL	4.39 × 10^−4^	1.29 × 10^−4^	3.395	0.002

### 3.6 Diagnostic Accuracy of S CHI3L1

Healthy controls had significantly lower median S CHI3L1 than patients (*p* < 0.001, Mann–Whitney test) (Table 6), indicating the potential for S CHI3L1 to serve as a diagnostic marker for distinguishing between healthy controls and patients.

**TABLE 6:** Comparison of healthy controls and all patients in CHI3L1 (µg/L).

	Min	Lower quartile	Median	Upper quartile	Max
Patients	18.2	36.5	58.7	129.1	745.4
Healthy controls	10.2	21.6	27.5	33.8	78.5
*p* value (Mann–Whitney test)	< 0.001				

According to the estimated reference range 14.44–63.11 µg/L (see Section 3.1), 39 out of 86 patients with known S CHI3L1 (45.3%) fell out of the estimated reference range, specifically demonstrating higher S CHI3L1 levels than the estimated upper bound. The application of the reference range 14.44–63.11 µg/L for the identification of patients yielded an accuracy rate of 72.7% (95% CI, 65.8%–78.8%), a sensitivity of 45.3% (95% CI, 34.6%–56.5%), and a specificity of 94.4% (95% CI, 88.3%–97.9%). A consideration of the selection of the upper bound of the reference range based on the patient's age (see Section 3.1) yields an accuracy of 67.0% (95% CI, 59.9%–73.6%), a sensitivity of 32.6% (95% CI, 22.8%–43.5%), and a specificity of 94.4% (95% CI, 88.3%–97.9%). The inferior accuracy measures observed in the latter approach may be attributed to the disparate age structure of the health controls. The median age of the healthy controls was significantly lower in comparison to all patients (*p* < 0.001; Mann–Whitney test). Therefore, caution is essential when applying the estimated reference ranges to the entire patient cohort.

**FIGURE 5:**
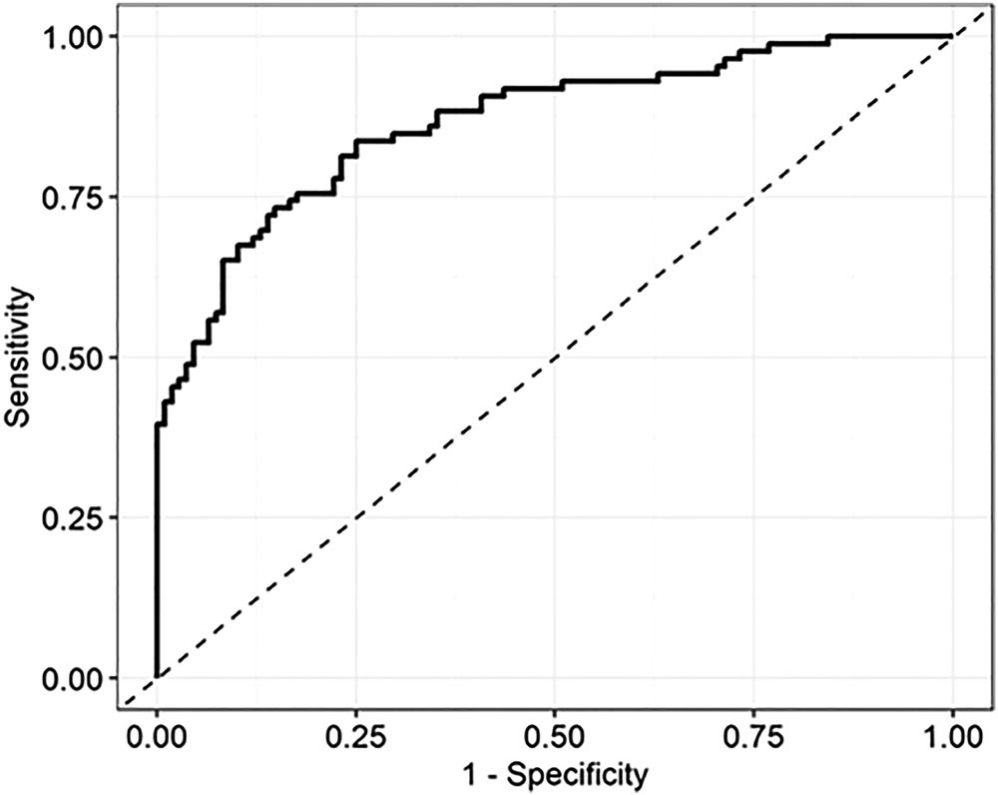
Analysis of the diagnostic accuracy of CHI3L1 with the ROC curve (AUC = 0.866, optimal cut‐off = 34.37 µg/L).

ROC analysis showed that the optimal cut‐off value of S CHI3L1 for identifying patients is 34.37 µg/L, with 78.9% accuracy (95% CI, 72.4%–84.4%), 81.4% sensitivity (95% CI, 71.6%–89.0%), and 76.9% specificity (95% CI, 67.8%–84.4%), which offer more promising measures of diagnostic accuracy than previous quantile‐based approaches (Figure 5).

## 4 Discussion

This study tested S CHI3L1 as a marker of inflammatory response accompanying neurodegeneration. We estimated the physiological serum levels of CHI3L1. Using the quantile regression model, we did not find a significant dependence of the lower bound (2.5% quantile) on sex or age, but there was a significant dependence of the upper bound (97.5% quantile) on age. The correlation between CSF levels of CHI3L1 and age has been published (Bonneh‐Barkay et al. 2010; Kušnierová et al. 2020); more recently, Sanfilippo et al. (2019) investigated the expression of CHI3L1 in distinct areas of the brain in males and females and proved correlations with age and even sex. As already written above, CHI3L1 is a relatively nonspecific mediator of inflammation involved in many organ dysfunctions (even smoking can elevate S CHI3L1) (Bara et al. 2012). The consequences of these processes grow with senescence, and the serum findings reflect them.

In accord with this, we proved a correlation between S CHI3L1 and age in the patient's cohort (groups 1–3). It has to be mentioned that these groups included elderly people burdened with more internal diseases, resulting in possible elevation of S CHI3L1.

A significant correlation was found between S CHI3L1 and S NFL in Groups 1 and 3 and between these biomarkers in all patients counted together. The connection between CHI3L1 and NFL has been studied in patients with multiple sclerosis (Cubas‐Núñez et al. 2021; Schneider et al. 2021), and Haji et al. (2022) found them to be correlated in the sera of patients with Parkinsonian syndrome. NfL is a relatively established biomarker of neuronal damage used in demyelinating and neurodegenerative diseases, where its levels reflect the severity of cognitive decline and dementia (Hradilek et al. 2023; Ning and Wang 2022; Novobilský et al. 2023). Although there is a correlation between S CHI3L1 and S NfL in patients with AD and Parkinsonism, unlike NfL, there are no significant differences in serum CHI3L1 concentrations among diagnostic groups. According to the literature, CHI3L1 levels in CSF can help differentiate AD from other dementias (Hampel et al. 2018; Janelidze et al. 2018; Muszyński et al. 2017).

Our comparison of S CHI3L1 and CSF NfL yielded different results, with a weak correlation found in all patients. Analogous results were observed between S CHI3L1 and CSF tTau in all groups. tTau reflects general neurodegeneration, similar to NfL (Holper et al. 2022). It is possible that if we were to compare these biomarkers in the same compartment, their mutual correlation would be more conclusive, as in the case of the S NfL.

We attempted to utilize common biomarkers of inflammation in patient samples, selecting serum CRP and WBC in blood. However, we did not observe a significant correlation between these markers and S CHI3L1 and S NfL. The potential of CRP in neurodegenerative diseases remains inconclusive, with some studies showing promise (Kravitz et al. 2009) and others yielding contrasting results (Ng et al. 2018).

The negative correlation between S CHI3L1 and cognitive impairment measured by MMSE has been published previously in patients with neuromyelitis optica (Jiang et al. 2023) and after hip fracture surgery (Zheng et al. 2023). According to Pase et al. (2024), higher plasmatic concentrations of CHI3L1 correlated with cognitive deterioration (measured by other cognitive batteries). A comparable correlation between CSF NfL and MMSE was observed by Dhiman et al. (2020) in patients with AD.

Our study has several limitations. First is the lack of unavailability of a more significant number of analyzed samples, both in the patient group and in the healthy controls. Furthermore, the composition of the patient groups was not entirely homogeneous.

Another limitation is CHI3L1 itself: it is a very nonspecific marker of inflammation, and its elevation in serum can be due to a wide variety of causes. We proved the dependency on age, but many other factors can play a role, for example, diabetes mellitus and cardiovascular diseases (Zhao et al. 2020).

A general limitation of research on neurodegenerative diseases is the uncertainty of diagnoses. Apart from AD, with its typical biomarker pattern and genetically determined disease, a definitive diagnosis can only be established postmortem. In addition, a combination of pathologies is sometimes detected at autopsy (Robinson et al. 2023).

## 5 Conclusion

According to our study, the ELISA method leads to promising results in measuring CHI3L1 in serum. CHI3L1 in serum can be used as a supportive biomarker in neurodegenerative diseases. However, as a stand‐alone marker, it is insufficient in diagnostics. In the emerging era of disease‐modifying anti‐amyloid therapy for AD, its correlation with cognitive decline may be utilized in the future as a biomarker for therapy monitoring, similar to how we use NfL in multiple sclerosis therapy.

## Author Contributions

**R. Novobilský**: conceptualization, investigation, writing – original draft, writing – review and editing, resources, project administration, methodology. **P. Bartova**: investigation, supervision, resources. **D. Stejskal**: supervision. **A. Kondé**: data curation, project administration, formal analysis, software, visualization, validation, methodology. **M. Bar**: supervision. **P. Kusnierova**: funding acquisition, methodology, writing – original draft, writing – review and editing, formal analysis, conceptualization, validation.

## Conflicts of Interest

The authors declare no conflicts of interest.

## Peer Review

The peer review history for this article is available at https://publons.com/publon/10.1002/brb3.70619

## Data Availability

The data that support the findings of this study are available on request from the corresponding author. The data are not publicly available due to privacy or ethical restrictions.
